# Evidence of substantial heterogeneity in the preventive effect of stricter alcohol policy environments in young Swiss men

**DOI:** 10.1192/j.eurpsy.2021.1885

**Published:** 2021-08-13

**Authors:** S. Foster, M. Mohler-Kuo

**Affiliations:** 1 Department Of Child And Adolescent Psychiatry And Psychotherapy (kjpp), University Hospital of Psychiatry Zurich, University of Zurich, Zurich, Switzerland; 2 La Source, School Of Nursing Sciences, HES-SO University of Applied Sciences and Arts of Western Switzerland, Lausanne, Switzerland

**Keywords:** alcohol drinking, alcohol policy, Young adults, machine learning

## Abstract

**Introduction:**

The alcohol policy environment was shown to exert a preventive effect on alcohol consumption. However, little is known about the heterogeneity of this effect.

**Objectives:**

To capture the extent of heterogeneity in the relationship between the strictness of alcohol policy environments and heavy drinking and to identify potential effect modifiers.

**Methods:**

Method: Cross-sectional data from 5986 young Swiss men participating in the cohort study on substance use risk factors (C-SURF) in Switzerland was analysed. Self-reported risky single-occasion drinking (RSOD, drinking 6 standard drinks or more on a single occasion at least monthly) in the past 12 months was the outcome of interest. A previously-used index of alcohol policy environment strictness across Swiss cantons was analysed in conjunction with 21 potential effect modifiers. Random forest machine learning and individual conditional expectations captured high-dimensional interaction effects and the heterogeneity induced by the interaction effects and identified potential effect modifiers.

**Results:**

Subject-specific absolute risk reductions ranged from 16.8% to -4.2%, with the latter implying a risk increase. Four prototypical subgroups were evident: “preventive” (alcohol policy environment decreased RSOD risk), “causative” (alcohol policy environment increased RSOD risk), “immune” (no effect due to low RSOD baseline risk), and “doomed” (no effect due to high RSOD baseline risk). Antisocial personality disorder and sensation seeking were major effect modifiers that reduced the preventive effect of stricter alcohol policy environments.

**Conclusions:**

Conclusion: Whereas stricter alcohol policy environments were associated with a reduced RSOD risk, adding selective prevention measures that target high-risk subpopulations is necessary.

**Disclosure:**

No significant relationships.

